# Comparative Transcriptome Sequencing Analysis of *Hirudo nipponia* in Different Growth Periods

**DOI:** 10.3389/fphys.2022.873831

**Published:** 2022-06-23

**Authors:** Xiaocong Ma, Xiuying Yan, Ren Ke, Huiquan Shan, Saif ur Rehman, Tong Feng, Yalin Zheng, Chen Chuang, Weiguan Zhou, Qingyou Liu, Jinghui Zheng

**Affiliations:** ^1^ Department of Cardiology, Ruikang Hospital Affiliated to Guangxi University of Chinese Medicine, Nanning, China; ^2^ State Key Laboratory for Conservation and Utilization of Subtropical Agro-bioresources, Guangxi University, Nanning, China; ^3^ Guangxi Medical University Cancer Hospital, Nanning, China; ^4^ Thai Natural Hirudin Co, Ltd., Bangkok, Thailand; ^5^ Guangdong Provincial Key Laboratory of Animal Molecular Design and Precise Breeding, School of Life Science and Engineering, Foshan University, Foshan, China

**Keywords:** Hirudo nipponia, different growth periods, transcription analysis, developmental, reproductive pathways

## Abstract

*Hirudo nipponia* is the only blood-sucking leech included in Chinese Pharmacopoeia having distinct features of anticoagulation, exorcizing blood stasis, and promoting menstruation. Despite such significant characteristics, very little is known about its molecular genetics and related physiological mechanisms. In this study, the transcriptomes of *H. nipponia* at three developmental stages (larvae, young, and adults), revealed a total of 1,348 differentially expressed genes (DEGs), 223 differentially expressed lncRNAs, and 88 novel mRNAs. A significant diverse gene expression patterns were observed at different developmental stages which were analyzed by differential gene expression trends, and the overall gene expression trends consist of three overall down-regulated trends, and two overall up-regulated trends. Furthermore, the GO and KEGG enrichment functional annotation analysis revealed that these DEGs were mainly associated with protein hydrolysis, signal transduction, energy metabolism, and lipid metabolism while growth, development, metabolism, and reproduction-related DEGs were also found. Additionally, real-time quantitative PCR results confirmed deep sequencing results based on the relative expression levels of nine randomly selected genes. This is the first transcriptome-based comprehensive study of *H. irudo nipponia* at different developmental stages which provided considerable deep understanding related to gene expression patterns and their relevant developmental pathways, neurodevelopmental and reproductive characteristics of the leech.

## Introduction

*Hirudo nipponia* (Annelida, Clitellata, and Hirudiniformes) is widely distributed in many regions of the globe (Liu et al., 2018). Physiologically, leeches are narrow, slightly flattened, cylindrical, and small-sized creatures used as raw material in Chinese traditional medicine with diverse functioning such as anticoagulation, exorcizing blood stasis, and promoting menstruation (Saglam, 2019). They are hermaphroditic where the male part matures first and then mates heterozygously, and oviparously (Ceylan, 2020). After hatching, leech seedlings moult once every 10 days, leeches grow for one age after one molt, 30–90 days is the optimum growth period for leeches, and it takes approximately 80 days to grow from seedling to adult size (Manav et al., 2019). However, *H. nipponia* has a prolonged maturation cycle with low reproduction ability as the *H. nipponia* begins to lay eggs in the second year. The average number of spawning cocoons of *H. nipponia* was the lowest compared with *Whitmania pigra Whitman* and *Hirudinaria Poecilobdella manillensis* (Ceylan, 2020).

*H. nipponia* as a traditional Chinese medicine contains hirudin which is the strongest thrombin natural inhibitor found so far (Straub et al., 2011; Liu et al., 2019) and it has unique properties of hypolipidemic, anti-tumor, anti-fibrotic, and anti-inflammatory. Furthermore, it has widely been used clinically to treat a variety of diseases such as hypertension, hyperlipidemia, cerebral infarction and malignancies (Sig et al., 2017; Pickrell et al., 2020; Wang et al., 2021). In addition, leeches have the ability to self-repair damage to the central nervous system and restore function, and are often used as model organisms in the field of neurobiology (Tasiemski and Salzet, 2017; Meiser et al., 2019; Heath-Heckman et al., 2021). In recent years, the number of wild leeches has gradually been decreased due to environmental destruction and increased hunting which results in high market demand but low supply. Thus, *H. nipponia* breeding at the farm level with selective traits is emerging everywhere all around the world (Chen et al., 2018; Yu et al., 2021).

Many studies have focused on the role of effective components in leech and their cultivation (Iwama et al., 2019; Wang et al., 2021). Recent advancements in high-throughput technologies provide an opportunity to dig out in-depth and comprehensive high quality leech genomes. Previously Liu et al. (2018) conducted a comparative transcriptomic study on three leech species including rod-shaped bull leech, broad-bodied golden leech and the cave mountain leech, and reported differentially expressed genes related to the olfactory transduction pathway among them. Moreover, another transcriptome-based study of the *H. nipponia* salivary gland identified a large number of anticoagulants, anti-inflammatory, antibacterial and anti-tumour proteins (Lu et al., 2018). But very little is reported related to the omics of *H. nipponia*, which is the major obstacle in the development and application of *H. nipponia* gene resources effectively. In this study, RNA-seq was used to sequence the transcriptome of *H. nipponia* at different developmental stages including larvae (L), young (Y), and adult (A) to provide a scientific basis for later research on the breeding, growth, and development of *H. nipponia* and related functional genes as well as the lncRNAs which are important for chromosomal modification, transcriptional activation, and interference.

## Materials and Methods

### Sample Collection

*H. nipponia* used in the experiment was collected from Qinzhou Zhanhong traditional Chinese medicine company. The samples were divided into a larval stage (within 10 days of hatching and have not sucked the blood), young insect stage (about 3 months after hatching and sucked blood three times) and adult stage (about 2 years after hatching, sucked blood more than six times and were adult and sexually mature) (Tan et al., 2002; Lu et al., 2018). The leeches were directly killed with scissors, then 24 samples were collected from the anterior and posterior segments from each of the larvae and young insects, along with adult oral sucker, gonad, tail sucker, and remaining insects. All the collected samples were placed in liquid nitrogen and stored at −80°C for further analysis. Meanwhile, a total of nine intact individuals, three each of larvae, young and adults, were taken for histological observation, fixed in 4% paraformaldehyde, and stored at 4°C. Three individuals from each group were taken as biological replicates.

### Histomorphological Analysis

The histological features of *H. nipponia* were observed through paraffin embedding, sectioning, hematoxylin, and eosin staining. The muscles’ ultra-morphology was observed by microscope (EVOS FL auto), and the images were collected via micrograph system (EVOS auto).

### cDNA Library Construction and Sequence Data Analysis

Total RNA was extracted from different parts of the *H. nipponia* at different developmental stages (L, Y, and A) using TRIzol reagent (Throme, United Sttaes). The RNA was purified using the RNeasy Mini Kit (Oiagen, Germany). The cDNA library was sent to Beijing Anuo Uda Gene Technology and sequenced using the Illumina HiSeq 4,000 platform (NEB, United Sttaes), which generated 150 bp paired-end reads. Each sample quality was checked using Fastp (v0.20.1) (Chen et al., 2018). After that HISAT2 (v2.2.1) (Kim et al., 2015) was used to map the reads from each sample to the reference genome and Samtools (v1.11) (Li et al., 2009) was employed to convert the sequence SAM files into BAM. StringTie (v2.1.4) (Pertea et al., 2015) was then applied to assemble and merge the transcripts from each sample. Gffcompare v0.11.2 software (Pertea and Pertea, 2020) was used to compare the merged transcripts to the reference annotation file in GTF, and StringTie was used to compare the transcripts to the reference annotationfile in GTF. StringTie was used to estimate transcript abundance using the option "-e-B-P20”. The Abundance results files were ending in ". balltown” and the prepDE.py was used to compare these files.

### Analysis of Differentially Expressed Genes

The FPKM (Fragments Per Kilobase of exon model per Million mapped fragments) method was used to calculate the expression of all genes in each sample. The differentially expressed genes (DEGs) between different groups were analyzed using DESeq2 software. Genes with FDR (false discovery rate) < 0.05 and |log2FC|>1 was set as a criterion to find the DEGs between two stages and Ggplot2 software was used to locate the DEGs.

### Sample Time Series Analysis

The Tcseq (time-course sequencing data analysis) software was used to analyze the DEGs at three-time points, and after clustering the patterns of DEGs were visualized at different developmental stages. The FDR value <0.05 and |log2FC|>1 was considered as the level of significance to determine the DEGs between the two stages.

### Functional Enrichment Analysis of DEGs

The functional annotation analysis of DEGs was done by GO (gene ontology) and KEGG (Kyoto Encyclopedia of Genes and genomes) using a cluster profiler (statistical analysis and visualization of gene and gene cluster functional maps). The value of FDR <0.05 was considered to be significantly enriched.

### Identification and Analysis of lncRNAs

Potential lncRNAs were screened by the following highly stringent criteria: 1) transcript length was not less than 200 nt; 2) transcripts were labeled as “i”, “u”, “324” and “x”; 3) the coding potential calculator (CPC2) score was “non-coding”; 4) transcripts containing an open reading frame (ORF) greater than 50 AA were removed; 5) transcripts were identified by alignment with the Swiss lncRNAs act as a cis-regulatory factor and can regulate the expression of adjacent genes. Furthermore, the co-expression networks of candidate lncRNAs and their upstream or downstream 10 kb mRNAs were constructed. Whereas, the ligation and enrichment were performed according to the position-frequency matrix.

### Synthesis of cDNA and Real-Time qPCR

We have selected nine genes randomly to validate the expression of RNA-Seq and the genes related to the growth and development of the *H. nipponia* as suggested by Shan et al. (2022). The polyubiquitinase gene (ubiquitin, *UBQ*) (Robledo et al., 2014; Peña et al., 2010) was selected as the internal reference gene and sequenced together with the validation gene used the oligo7 designed (Supplementary Table S1), with primers synthesized by Sangon Biotech (Shanghai, China). The RNA was transformed into cDNA using the Hiscript III Reverse Transcription Kit (Vazyme, Nanjing, China), and fluorescent quantitative PCR was performed on an ABI 7500 (Thermo, MA, United Sttaes) using the AceQ qPCR SYBR Green Master Mix system (Vazyme, Nanjing, China). The relative quantitative 2^−ΔΔCt^ method was used to analyze genes expression (Xu et al., 2021).

## Results

### Developmental Features and Histological Observations of *H. nipponia* at Different Developmental Stages

**FIGURE 1:**
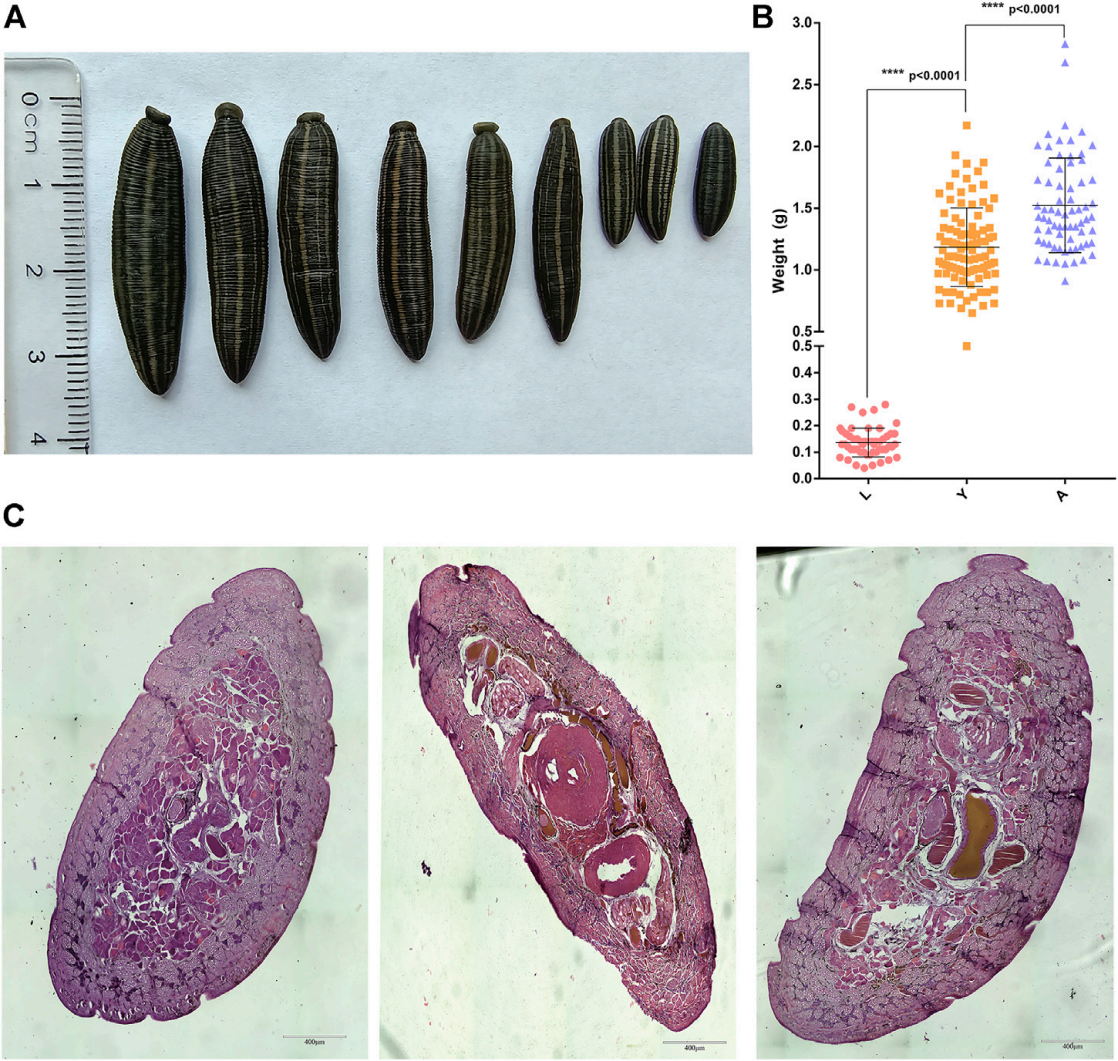
Phenotypic data of *H. nipponia*. **(A)** Observation on the body shape of *H. nipponia* in different periods. **(B)** Weight statistics of *H. nipponia* in different periods. **(C)** Histological observation of *H. nipponia* in different periods.

The formaldehyde-fixed *H. nipponia* at different developmental stages such as larvae (L), young (Y), and adults (A) are shown in Figure 1A. The calculated bodyweight of *H. nipponia* larvae (0.1362 ± 0.0075 g), young insects (1.184 ± 0.031 g), and adults (1.524 ± 0.045 g), were found significantly different in three developmental stages (*p* < 0.0001) as presented in Figure 1B. Additionally, the results of paraffin sections at different growth stages showed that there was a significantly higher ratio of muscle bundle tissue in the sections of adult worms as compared to larvae and young leech (Figure 1C).

### RNA Sequencing of *H. nipponia*

We constructed a total of 24 cDNA libraries of *H. nipponia* at different growth stages (HNLB1, HNLB2, HNLB3, HNLF1, HNLF2, HNLF3, HNYB1, HNYB2, HNYB3, HNYF1, HNYF2, HNYF3, HNO1, HNO2, HNO3, HNG1, HNG2, HNG3 (HNP1, HNP2, HNP3, HNB1, HNB2, and HNB3) as shown in Supplementary Table S1. Three samples were taken from each of the larval, young, and adult stages (HNL1, HNL2, HNL3, HNY1, HNY2, HNY3, HNA1, HNA2, and HNA3). A total of 541,124,257 raw reads were generated from 24 libraries and after filtering the adapters, N ratios greater than 10%, basic reads, and low-quality reads, about 517805628 clean reads were obtained. The GC content of all the samples ranging between 41% and 45% (Supplementary Table S2). These results indicate that the sequencing data are of good quality and meet the requirements for subsequent analysis. The specific data are listed in Supplementary Table S2.

**FIGURE 2:**
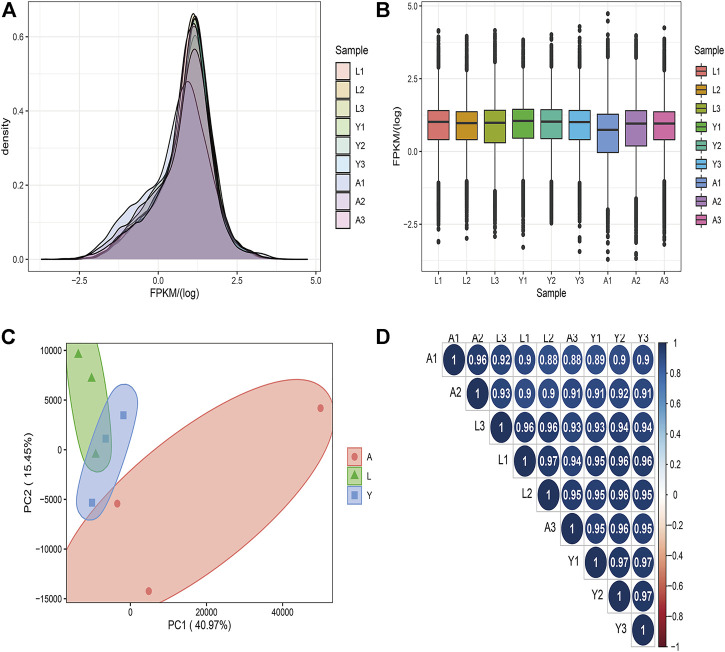
The mRNA expression analysis. **(A)** The density distribution of mRNAs was according to log10 (FPKM). **(B)** The 9 Samples expression (L1, L2, L3, Y1, Y2, Y3, A1, A2, and A3) violin plot, which was replaced by log10 (FPKM). **(C)** The PCA distribution of 9 samples. **(D)** The sample relationship cluster analysis.

A total of 20,430 mRNAs were detected by RNA-seq, of which 88 were novel. The transcriptional expression of mRNAs was expressed as FPKM values and the FPKM distribution of all mRNAs is shown in Figure 2A, while the gene expression of the different samples is shown in a box plot (Figure 2B). To find the “main” elements and structures in the data, the complex sample composition relationship is reflected in the two eigenvalues of horizontal and vertical coordinates, which were helpful to explore the distance relationship between samples. Moreover, based on a permutation multivariate analysis of variance (PERMANOVA) we obtained *p*-value = 0.04 (*p* < 0.05) and then the nine samples were further divided into three sections, showing a tendency to cluster within the same group and a significant separation between the different groups (Figure 2C) and a relational clustering graph (Figure 2D) was constructed to show the correlation between each sample. We observed that the distribution of genes expression data for the samples we acquired was relatively consistent, and the PCA analysis results exhibited that the different subgroups can be more clearly distinguished from one another, and that intra-group correlations are high, providing reliable data for subsequent analysis.

### Differential Gene Expression Analysis

**FIGURE 3:**
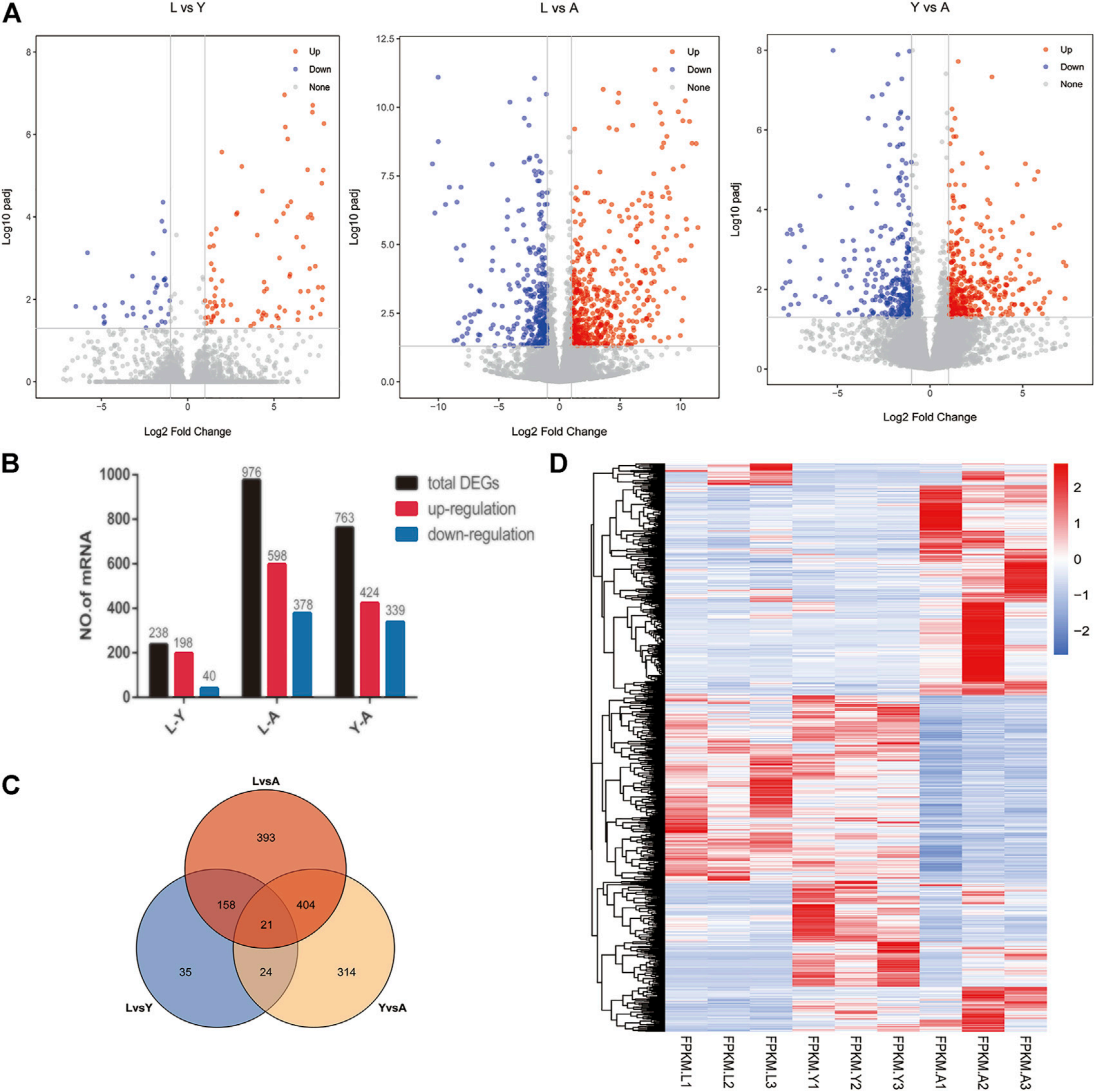
Visual analysis of expression differences between contrasts at different growth periods. **(A)** The barplot of the DEGs in three comparisons Volcano plot displaying DEGs between HNL vs. HNY, HNL vs. HNA, HNY vs. HNA stages. **(B)** Genetic statistics for differences between growth periods. **(C)** The ven plots of differential genes in different growth periods. **(D)** Heat map of genes in different growth periods.

We used FDR value <0.05 and |log2FC| > 1 as criteria for screening DEGs by pairwise comparisons of three different growth stages of the *H. nipponia*. We obtained 238 DEGs in LvsY, 976 DEGs in LvsA, and 763 DEGs in YvsA (Figures 3A,B). We plotted the VEEN diagram for DEGs of these different growth periods (Figure 3C). In total, we obtained 1,348 DEGs, and the expression of these genes was shown in the heat map using cluster analysis (Figure 3D). The result showed that the adult stage had more ratio of DEGs and active expression than that of larvae and young, while on the other hand, the differential genes between larvae and young worms were fewer but more stable. Thus, it was observed that the expression of different genes in *H. nipponia* differed significantly at different developmental periods.

### Sample Time Series Analysis of DEGs

**FIGURE 4:**
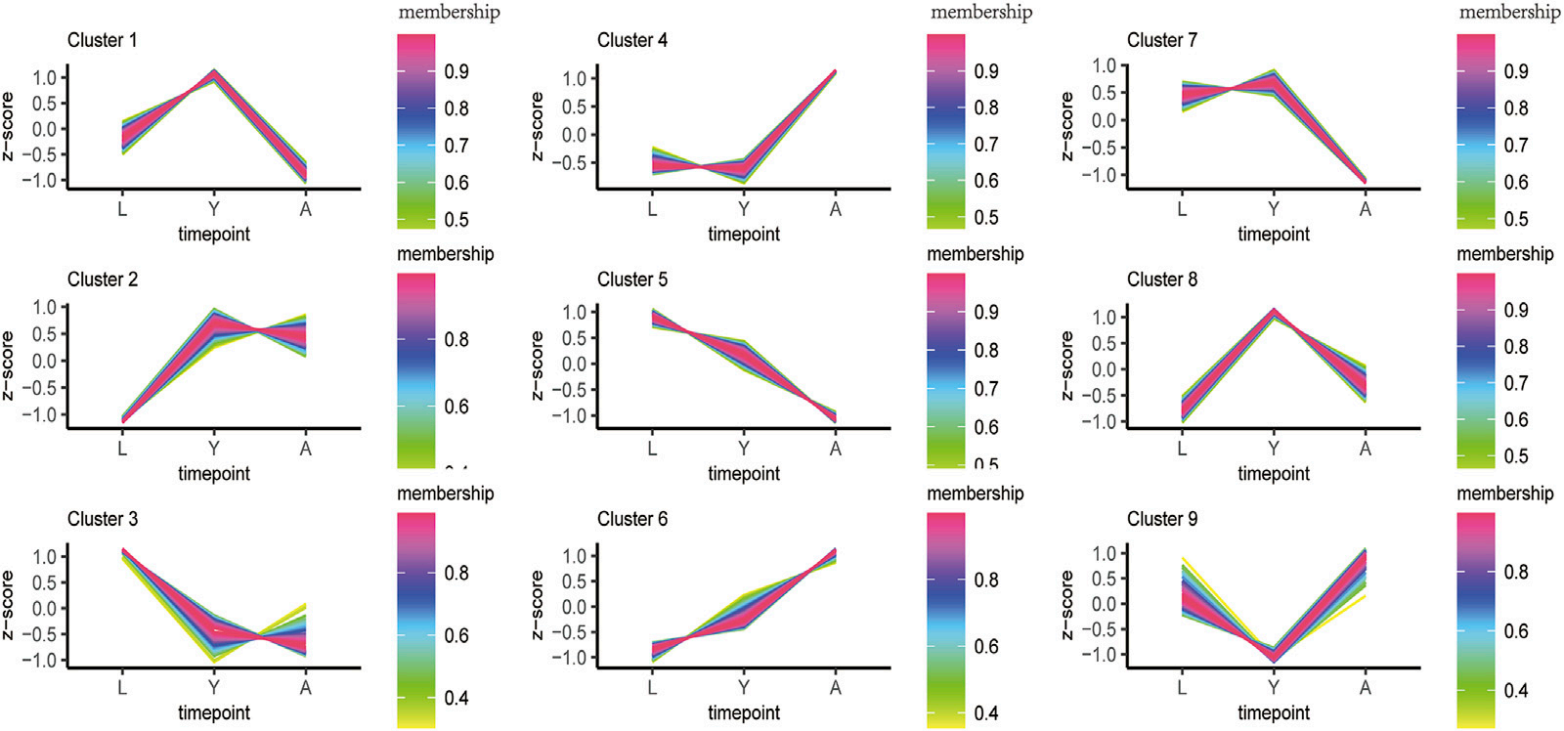
The sample time series analysis of DEGs. [Note: The horizontal axis represents different growth periods, and the vertical axis represents normalized gene expression].

In order to fully reveal the growth and development of *H. nipponia* at different time points, we analyzed the expression trends of differential genes using FDR <0.05 and |log2FC|>1 as a level of significance. The overall gene expression trends included three overall down-regulated trends (cluster1, 5, and 7), two overall up-regulated trends (cluster 4 and 6), and three highest trends of DEG expression in HNY (cluster1, 2, and 8) (Figure 4). The changes in DEGs and their trend enrichment indicated significant differences in gene expression profiles at different developmental stages, effectively revealing the gene expression status of *H. nipponia* during development.

### GO and KEGG Enrichment Analysis of DEGs

**FIGURE 5:**
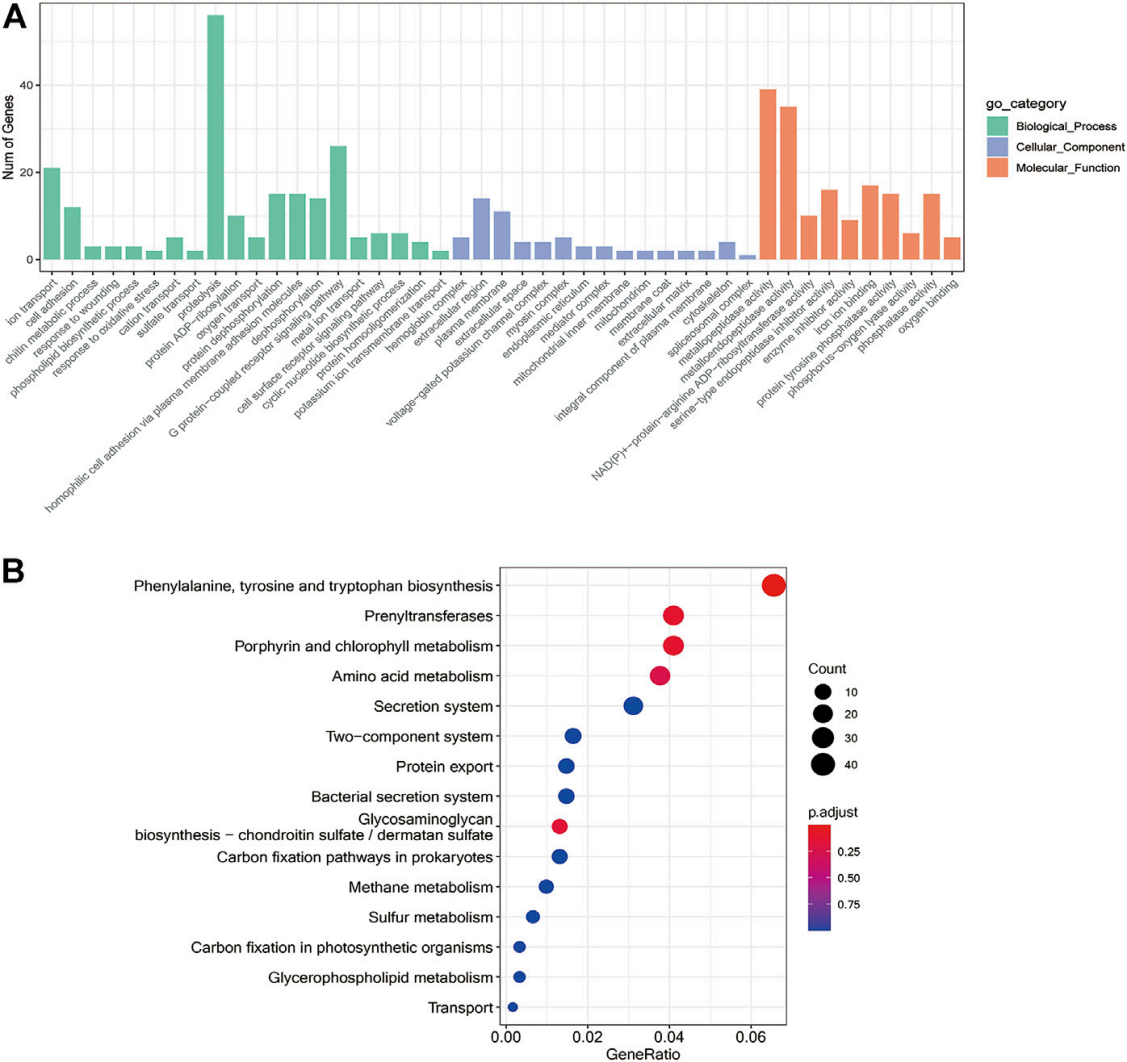
Differential gene enrichment analysis. **(A)** GO functional enrichment analysis differential genes. **(B)** Enrichment analysis of the KEGG pathway for differential genes.

To further unravel the functions of differentially expressed genes, GO analysis was performed and grouped the functional annotation into three categories: molecular function, cellular component, and biological process (Figure 5A). The DEGs were mainly enriched in 71 biological processes, 23 cellular components, and 130 molecular functions. The molecular functions of DEGs were found to be associated with ion transport, cell adhesion, proteolysis, and G protein-coupled receptor signaling pathway, while cellular components were plasma membrane and hemoglobin complex and the biological processes included protein dephosphorylation, homophilic cell adhesion via plasma membrane adhesion molecules, oxygen transport, response to wounding and cyclic nucleotide biosynthetic process. The KEGG enrichment is mainly related to metabolism, for example, amino acid metabolism such as Phenylalanine, tyrosine and tryptophan biosynthesis, and energy metabolism like glycerophospholipid metabolism, carbon sequestration in photosynthetic organisms, and carbon fixation pathways in prokaryotes as shown in Figure 5B. In addition, GO and KEGG profiles of differentially expressed genes were compared between the two *H. nipponia* at different periods as shown in Supplementary Figure S1, S2.

### Analysis of Differentially Expressed lncRNAs (DElncRNAs)

**FIGURE 6:**
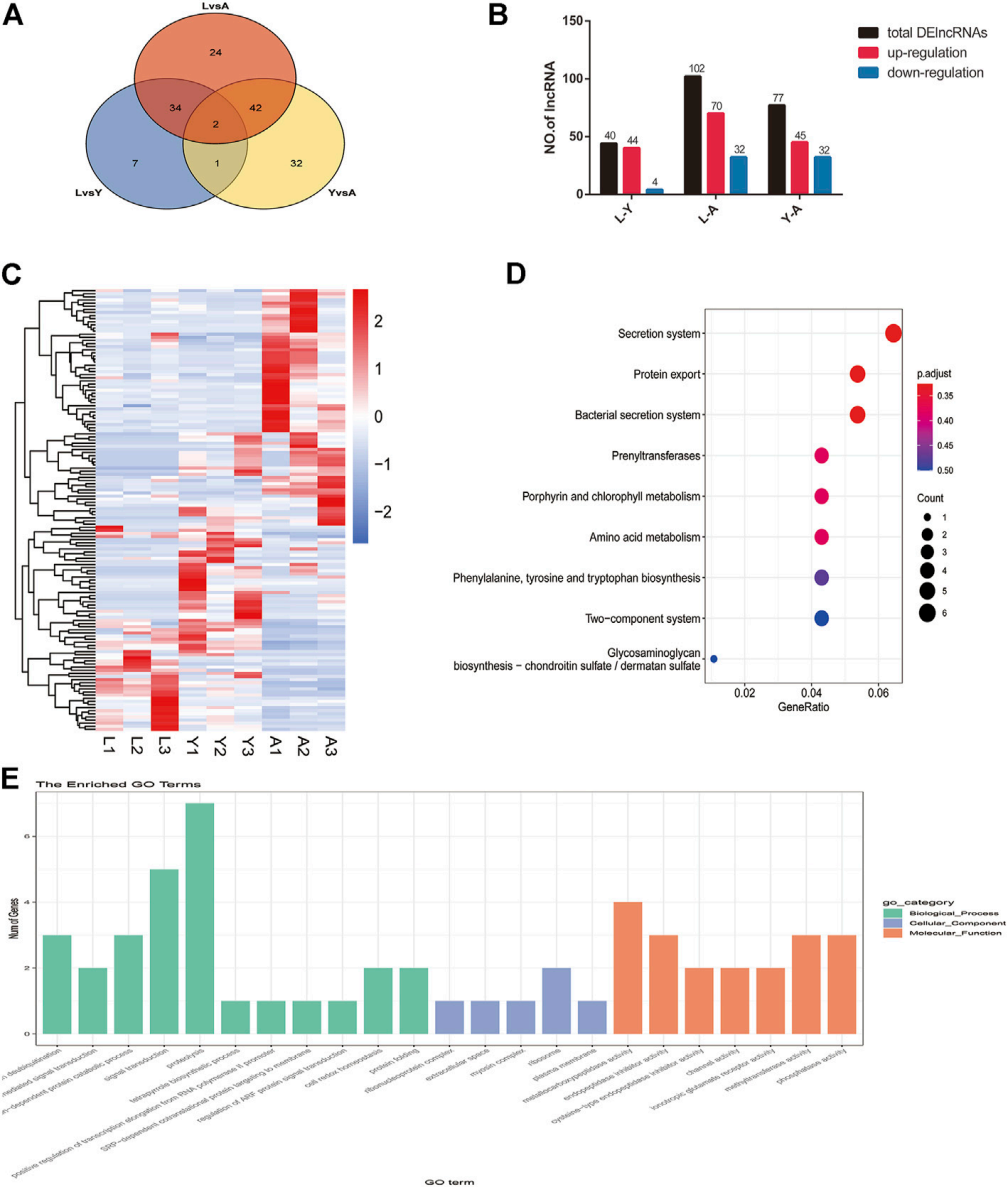
Screening and enrichment analysis of DelncRNAs in *H. nipponia* at different growth stages. **(A)** DelncRNA statistics at different growth stages. **(B)** Venn diagram of DElncRNAs. **(C)** DelncRNA clustering analysis. **(D)** KEGG analysis for revealing the role of predicted DelncRNA target genes. **(E)** GO enrichment plot showing GO analysis of predicted DelncRNA target genes.

Furthermore, we compared two-by-two differences between the three different growth stages of *H. nipponia* for screening DelncRNAs by using FDR <0.05 and |log2FC|>1 as a criterion and in total, 223 DelncRNAs were obtained (Figure 6A) of these, 44 DelncRNAs were found in LvsY, 102 in LvsA, and 77 in YvsA (Figure 6B). The clustering analysis of these DelncRNAs is presented in a heat map (Figure 6C). Additionally, the GO enrichment analysis showed that DelncRNA target genes were mainly enriched in protein deubiquitination, ribosome, and metallo-carboxypeptidase activities (Figure 6D) whereas, the KEGG pathway analysis exhibited that the DelncRNA target genes were mainly correlated to protein secretion, bacterial secretion system, and secretion system (Figure 6E).

### The Expression of Important Genes Related to Growth and Development

**FIGURE 7:**
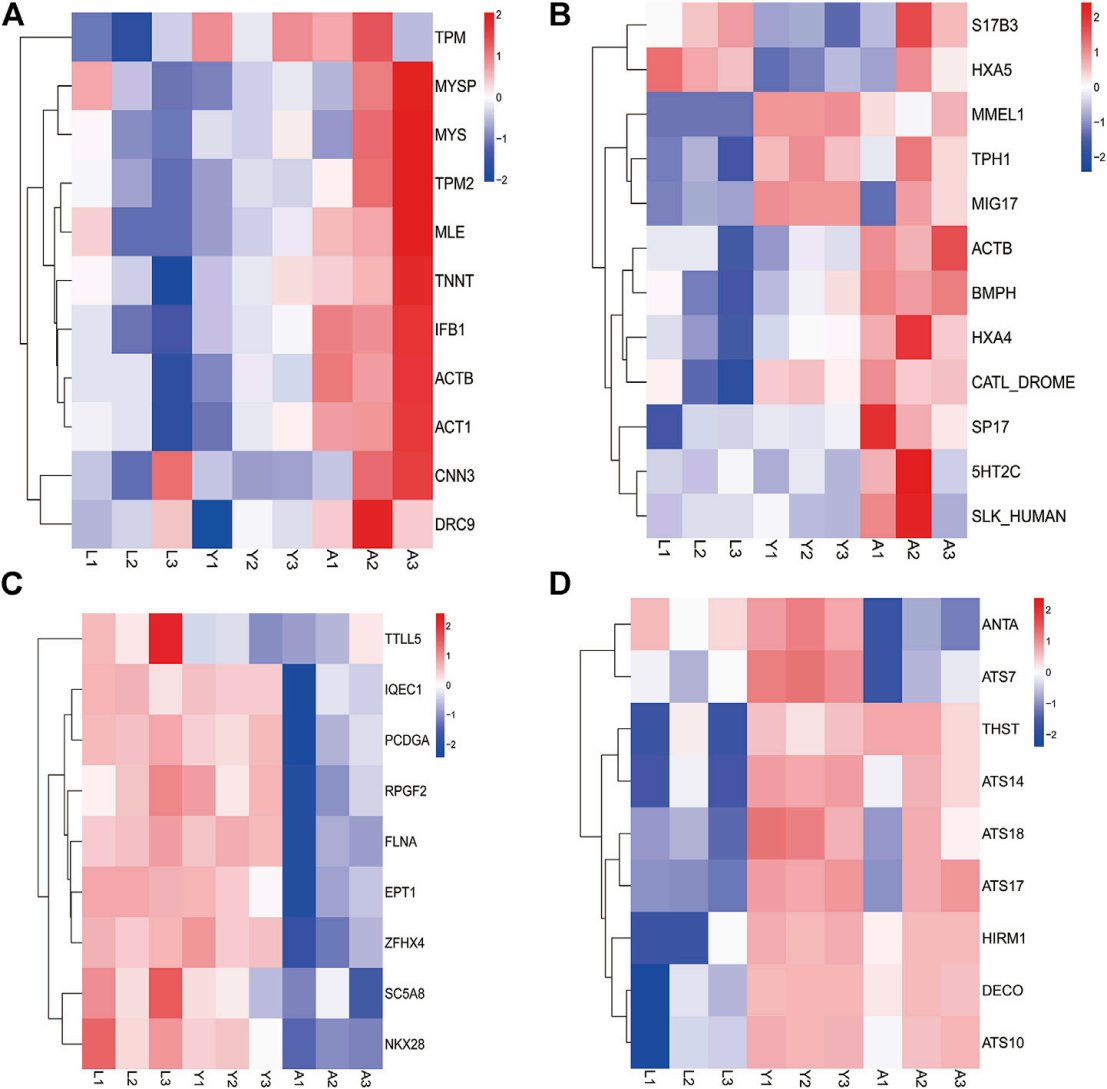
The expression of important genes related to growth and development. **(A)** Muscle development-related genes. **(B)** Reproduction-related genes. **(C)** Neurodevelopment-related genes. **(D)** Anticoagulation-related genes.

Furthermore, the DEGs were evaluated and several genes associated with leech growth and development including muscle development-related genes such as promyosin, myosin heavy chain, myosin light chain, actin, etc. were determined which showed a progressive increase in gene expression as the leech developed (Figure 7A). However, the genes involved in gonadal development or regulation of egg production in leeches like surface protein (*SPA17*), IQ motif containing G (*IQCG*), *β*-actin *(ACTB*), 5-hydroxytryptamine (*5-HT*), and HOX gene family, etc. were highly expressed in the adult stage (Figure 7B). Similarly, the genes associated with neurodevelopment e.g., Tubulin Tyrosine Ligase Like 5 (*TTLL5*), Filamin-A (*FLNA*), Protocadherin Gamma Subfamily A10 (*PCDHGA10*), Solute Carrier Family 5 Member 8 (SLC5A8) and NK2 Homeobox 8 *(NKX2-8)*, etc. were expressed in all growth period but were highly expressed in the larval stage (Figure 7C). On the other hand, in young worms, the anticoagulation-related genes Antistasin (ANTA), a disintegrin and metalloproteinase with thrombospondin motif family (*ADAMTS*), therostasin (*THST*), and decorsin (*DECO*) etc. were highly expressed (Figure 7D).

### Confirmation of Gene Expression With Quantitative PCR

**FIGURE 8:**
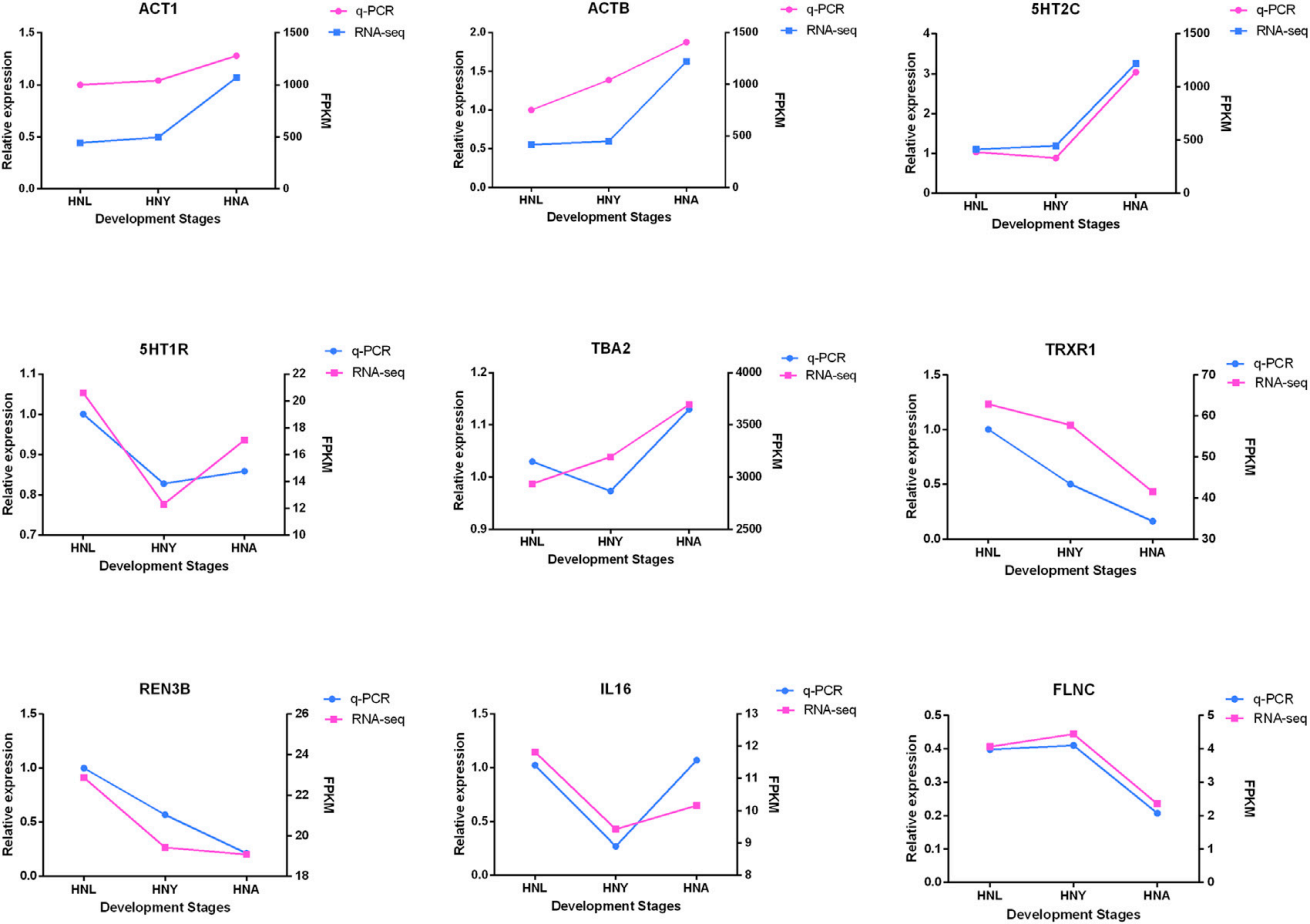
The validation of candidate genes. Red means q-PCR and blue means RNA-seq.

To verify the expression of genes from transcriptome sequencing data, we randomly selected 9 genes, including 5-Hydroxytryptamine Receptor 7 (*5HT1R*), *ACTB*, and *UPF3B* Regulator of Nonsense Mediated MRNA Decay (*REN3B*), etc. for RT-qPCR validation. The results show that the expression trends of the genes verified by RT-PCR are in line with the RNA-seq trends (Figure 8), and indicated that the obtained transcriptome sequencing data are highly reliable.

## Discussion

In this study, we reported transcriptomic analyses from three different developmental stages (L, Y, and A) of *H. nipponia*, thus significantly expanding the genetic resources available for the leech. Our study also provides a new theoretical basis for studying the molecular genetics-based physiological mechanisms involved in the development of *H. nipponia* which ultimately benefits to improve the aquaculture privilege of *H. nipponia*. Based on growth characteristics, the leeches have categorized into three growth or developmental stages such as the larva that have not sucked blood for about 10 days after hatching, young worm included those that have sucked blood three times after hatching for about 2 months, and the adults sucked blood more than six times after 2 years of hatching and are sexually mature. Totally, 1,348 DEGs and 223 DelncRNAs were found in three growth periods, among them, the highest number of differential genes were identified in larval and adult leeches, while the lowest number of differential genes were figured out in young and adult leeches, which may be related to the fact that 3-month-old young leeches are similar in size to adult leeches (Tan et al., 2002; Lu et al., 2018). Furthermore, the GO and KEGG enrichment analyses showed that differential genes expression patterns were mainly associated with protein hydrolysis, enzyme activity, metabolism, and cell growth and development. As leeches grow rapidly during the young worm period, requiring large amounts of food intake and energy through synthesis, digestion, and metabolism of food, our data show that genes associated with digestion and metabolism were highly expressed in the young worm stage. In addition, genes encoding enzymes with protease activity (metalloproteases and thiol proteases) were found upregulated in young worms, consistent with studies showing that the non-reproductive stage (often referred to as the feeding stage) exhibits more aggressive feeding behavior than the adult stage (Zhao et al., 2021).

lncRNAs are long-stranded non-coding RNAs that are greater than 200 nt in length and do not have a coding role in living organisms. Although lncRNAs do not encode proteins but they have a crucial role in life activities. It has been demonstrated that lncRNAs are involved in X chromosome inactivation, chromosomal remodeling, transcriptional activation and interference as well as growth and development, metabolism and differentiation, and tumor production. Compared to other species, the study on leech lncRNAs is not well documented. In this study, during three developmental periods (L, Y, and A) a total of 223 differentially expressed lncRNA molecules were identified. Further analysis of these lncRNA molecules indicated that these lncRNA molecules are likely to play an important role in the growth and development of *H. nipponia*. Our study data provide new insights into the regulatory mechanisms of lncRNAs in the growth and development of *H. nipponia*.

Typically, actin, *Ef1a*, and *GAPDH* are used as internal reference genes for RT-qPCR testing, however we selected *UBQ* as internal reference gene. This is because *β*-actin or *GAPDH* are differentially expressed genes according to the results of our RNA-seq sequencing datat therefore we cannot use *β*-actin or *GAPDH* as internal reference genes for qRT-PCR analysis. Since there is lack of literature on the selection of internal reference genes in *H. nipponia,* we looked to other species and found that *UBQ* could be used as a regular internal reference gene (Robledo et al., 2014; Peña et al., 2010) and was not a differentially expressed gene in our RNA-seq data, so it was selected as an internal reference gene.

Previous studies have shown that the degree of muscular growth has changed significantly at different growth stages along with the underlying mechanisms regulating muscle growth might differ (Li et al., 2021). Our results indicated that the genes attributing muscular development were highly expressed and showed a tendency of upregulation as the leech body develops. The muscle-building block protein of annelids, paramyosin (*MYSP*), plays a key function in the muscular tension contraction process (Sonobe et al., 2016; Silva et al., 2020). In addition to performing basic contractile functions, proto-myosin (*TPM*) is the actin-binding protein in muscle filaments is also involved in many vital activities in the body, such as cytoplasmic division and signal transduction. It can bind helically to actin filaments and regulate interactions with myosin (Zhang et al., 2018; Jurewicz et al., 2020; Pavadai et al., 2020). Actin is an important member of the actin family that controls almost all forms of cell and organelle motility which also regulates growth, development, bioregulation, and repair (Hammer et al., 2019; Kadzik et al., 2020; Liu et al., 2020). Myosins consist of the myosin heavy chain (*MYH*) and the myosin light chain. The *MYH* family is one of the key members in the regulation of muscle development and affects muscle production and repair after injury, mainly through the regulation of cell proliferation and differentiation (Pan et al., 2016; Squire, 2019; Hill et al., 2021).

Leeches are typically hermaphroditic, with the male gonads maturing first than female gonads. Compared to other species of leeches, *H. nipponia* has low reproductive performance. Though analyzing the DEGs, we screened the genes or proteins associated with gonadal development or oogenesis. For example, *SPA17, IQCG, ACTB, 5-HT*, and the *HOX* gene family. The *IQCG* is also involved in spermatogenesis and is a key regulator of cilia/flagellar motility and is associated with sperm motility (Harris et al., 2014; Gonzalez-Gutierrez et al., 2021). Additionally, the studies have also reported that *β*-actin is potentially involved in sperm maturation, sperm excretion, and the ability to maintain sperm motility. Early studies on mammalian (guinea pig) sperm showed that actin was involved in sperm motility and random severance of actin filaments inhibited the flagella motility (Yenia, et al., 2007). Moreover, comparative proteomic study on normal and weak spermatozoa revealed that reduced expression of *β*-actin results in feeble spermatozoa (Martinez-Heredia et al., 2008). Similarly, reports on ovarian development have shown that *5-HT* significantly play an important biological role in gonadal maturation and serial egg production (Kondo et al., 2017). The *HOX* gene is a family of regulatory molecules encoding highly conserved transcription factors whose expression is regulated by sex steroids, which are significantly involved in reproductive tract development, endometrial cycle growth, and embryo implantation (Du and Taylor, 2015; Cohen et al., 2020).

Furthermore, the leech is a fascinating model organism for researching brain regeneration mechanisms because of its capacity to regenerate the central nervous system (CNS) throughout its life cycle (Meriaux et al., 2011; Lu et al., 2018). Second, the leech’s CNS neurons are constantly expanding, implying that the process through which the leech develops and promotes synaptic connectivity may never be turned off. In our study, genes associated with neural development such as *TTLL5, FLNA, PCDHGA10, SLC5A8,* and *NKX2-8* were expressed in all three developmental stages but were highly expressed in the larval stage. These genes are thought to have a major role in the formation of certain synaptic connections as well as the maintenance of neuronal energy status and function. They are regulated in neurons which re-establish axonal and synaptic connections. In addition to hirustasin, other anticoagulant genes including *ANTA, ADAMTS, THST,* and *DECO* were also expressed in the leech, and they were highly expressed in young leeches. Hirustasin and *THST* inhibited trypsin, chymotrypsin, histone G and tissue kinase-releasing enzyme, but not coagulation factor *Xa* activity (Kwak et al., 2019). In contrast, *ANTA* is a potent inhibitor of Factor *Xa*. Additionally, *DECO* inhibits the interaction of fibrinogen with platelet receptors expressed, on the glycoprotein IIb-IIIa complex. It prevents blood clotting when feeding and/or storing ingested blood (El-Obeid and Al-Badr, 1982). Adult leeches are frequently used as medicine in production applications and their anticoagulant features make it worthwhile to utilize leeches in medicine, like *H. nipponia*, which is commonly used for the treatment of various ailments (Koeppen et al., 2019; Singh et al., 2020) Our findings indicated that the anticoagulant gene is highly expressed in young leech suggesting the possibility that young leeches are more effective in medicine while further experimental verification of the exact function is needed.

## Conclusion

In this study, the transcriptome of *H. nipponia* was systematically analyzed in three different developmental stages. Trait and histological observations revealed that adult leeches had significantly higher body length and body weight and a significantly higher number of myenteric tissues than larval and young leeches. Transcriptome analysis revealed that the number of DEGs was highest in L vs. A; DEGs enrichment analysis was mainly related to metabolic pathways; genes related to muscle development, genes related to gonadal development or oviposition regulation and genes related to neural development were significantly period-specific, indicating that these changes were associated with leech growth and development. In addition, we found that hirudin and anticoagulation-related genes were highly expressed in the young stage.

## Data Availability

The original contributions presented in the study are included in the article/Supplementary Material, further inquiries can be directed to the corresponding authors.
