# Supplementary material for: Comparative Transcriptome Sequencing Analysis of Hirudo nipponia in Different Growth Periods
Source: Front Physiol. 2022 Jun 23;13:873831. doi: 10.3389/fphys.2022.873831 (PMC9259933; doi:10.3389/fphys.2022.873831)
Supplement: Supplementary file 1 [file DataSheet1.ZIP › Supplementary Materials/Table S1-S2.docx]

Table S1 Sample number

| Sample name | Sample markings |
| --- | --- |
| *Hirudo nipponia* oral sucker 1 | HNO1 |
| *Hirudo nipponia* oral sucker 2 | HNO2 |
| *Hirudo nipponia* oral sucker 3 | HNO3 |
| *Hirudo nipponia* genital gland 1 | HNG1 |
| *Hirudo nipponia* genital gland 2 | HNG2 |
| *Hirudo nipponia* genital gland 3 | HNG3 |
| *Hirudo nipponia* body 1 | HNB1 |
| *Hirudo nipponia* body 2 | HNB2 |
| *Hirudo nipponia* body 3 | HNB3 |
| *Hirudo nipponia* posterior sucker 1 | HNP1 |
| *Hirudo nipponia* posterior sucker 2 | HNP2 |
| *Hirudo nipponia* larva front 1 | HNLF1 |
| *Hirudo nipponia* larva front 2 | HNLF2 |
| *Hirudo nipponia* larva front 3 | HNLF3 |
| *Hirudo nipponia* larva back 1 | HNLB1 |
| *Hirudo nipponia* larva back 2 | HNLB2 |
| *Hirudo nipponia* larva back 3 | HNLB3 |
| *Hirudo nipponia* young front 1 | HNYF1 |
| *Hirudo nipponia* young front 2 | HNYF2 |
| *Hirudo nipponia* young front 3 | HNYF3 |
| *Hirudo nipponia* young back 1 | HNYB1 |
| *Hirudo nipponia* young back 2 | HNYB2 |
| *Hirudo nipponia y*oung back 3 | HNYB3 |

Table S2 Summary statistics of *Hirudo nipponia* transcriptome sequencing

| Dataset name | Raw reads | Clean reads | GC content | avg_sequence_length |
| --- | --- | --- | --- | --- |
| HNB1 | 22861205 | 22859929 | 44 | 149.9192 |
| HNB2 | 22522569 | 22521770 | 42 | 149.7963 |
| HNB3 | 21167137 | 21166173 | 43 | 149.8346 |
| HNG1 | 23297376 | / | 43 | 149.759 |
| HNG2 | 23297376 | 23296330 | 43 | 149.759 |
| HNG3 | 22783646 | 22782068 | 42 | 149.8835 |
| HNLB1 | 22333657 | 22332824 | 44 | 149.7919 |
| HNLB2 | 22274479 | 22273449 | 45 | 149.7734 |
| HNLB3 | 22755945 | 22754982 | 44 | 149.8208 |
| HNLF1 | 22483696 | 22483048 | 43 | 149.7944 |
| HNLF2 | 23660482 | 23659518 | 44 | 149.7686 |
| HNLF3 | 21711954 | 21711309 | 44 | 149.8045 |
| HNO1 | 23024727 | 23023566 | 44 | 149.9142 |
| HNO2 | 22519063 | 22518396 | 42 | 149.7088 |
| HNO3 | 23115911 | 23115287 | 42 | 149.807 |
| HNP1 | 20369024 | 20367936 | 44 | 149.8747 |
| HNP2 | 22625716 | 22624682 | 41 | 149.8698 |
| HNP3 | 22030817 | 22029737 | 41 | 149.8826 |
| HNYB1 | 22675116 | 22674491 | 43 | 149.7699 |
| HNYB2 | 22419524 | 22418633 | 44 | 149.7752 |
| HNYB3 | 22997036 | 22996301 | 44 | 149.7786 |
| HNYF1 | 23266207 | 23265301 | 43 | 149.7238 |
| HNYF2 | 22308856 | 22308114 | 43 | 149.7769 |
| HNYF3 | 22622738 | 22621784 | 43 | 149.689 |


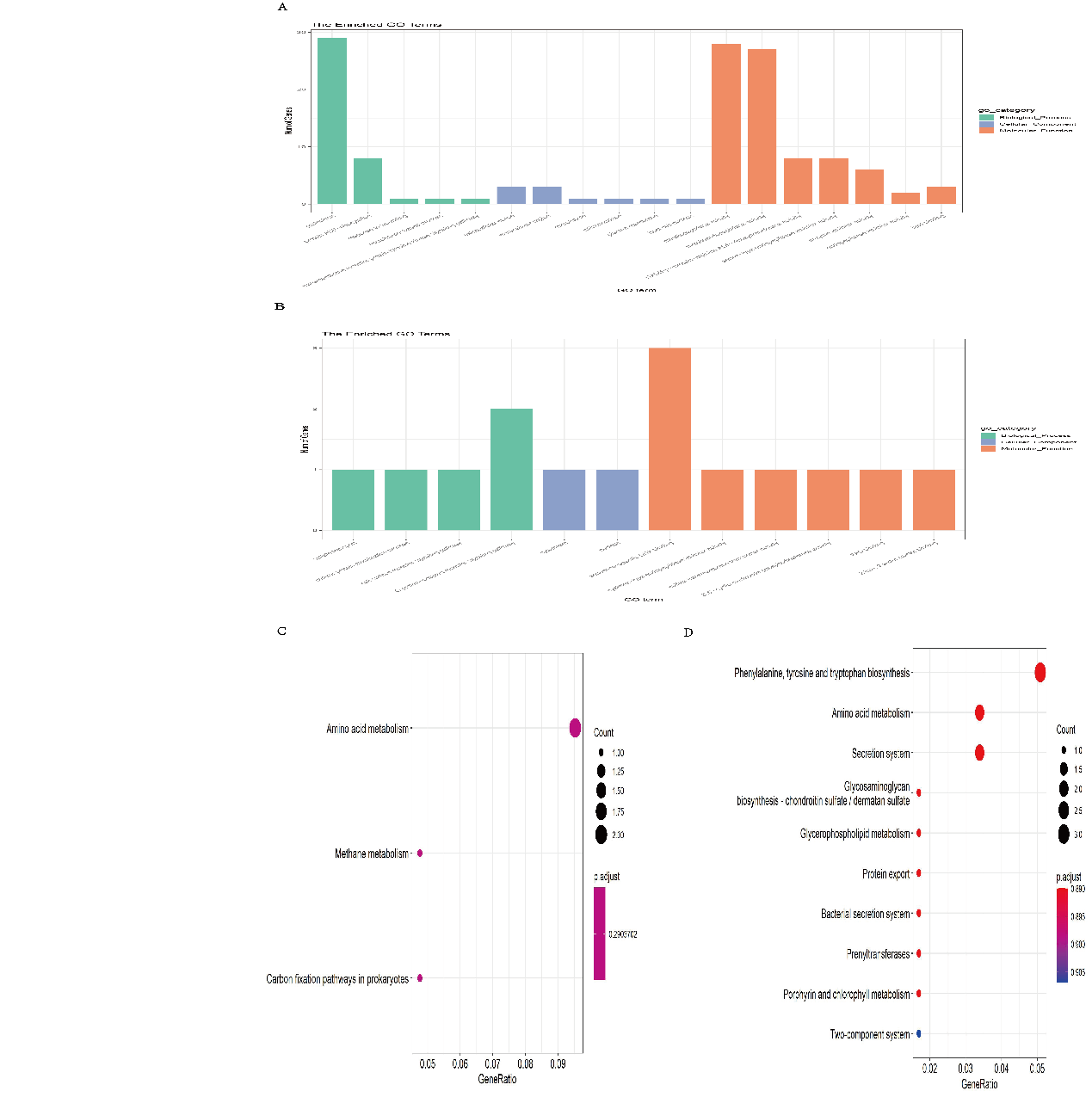


Figure S2 The DEGs enrichment analysis in Y vs A period

GO enrichment analysis of (A) up-regulated (B) down-regulated differential genes during Y vs A period; KEGG enrichment analysis of (C) up-regulated (D) down-regulated differential expressed genes during Y vs A period


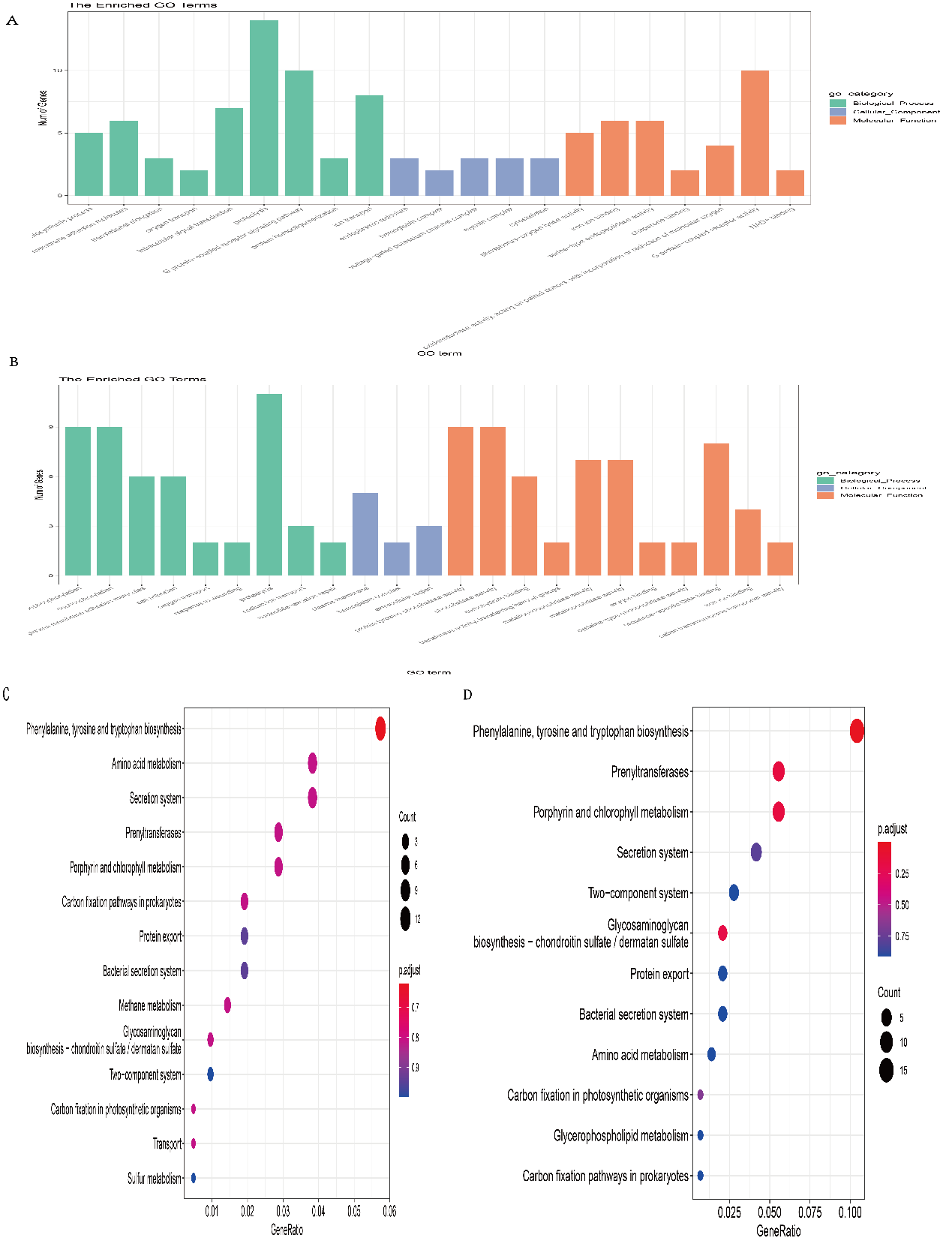


Figure S2 The DEGs enrichment analysis in Y vs A period

GO enrichment analysis of (A) up-regulated (B) down-regulated differential genes during Y vs A period; KEGG enrichment analysis of (C) up-regulated (D) down-regulated differential expressed genes during Y vs A period
